# Acute Mental Discomfort Associated with Suicide Behavior in a Clinical Sample of Patients with Affective Disorders: Ascertaining Critical Variables Using Artificial Intelligence Tools

**DOI:** 10.3389/fpsyt.2017.00007

**Published:** 2017-02-02

**Authors:** Susana Morales, Jorge Barros, Orietta Echávarri, Fabián García, Alex Osses, Claudia Moya, María Paz Maino, Ronit Fischman, Catalina Núñez, Tita Szmulewicz, Alemka Tomicic

**Affiliations:** ^1^Facultad de Medicina, Departamento de Psiquiatría, Depression and Suicidality Research Group, Pontificia Universidad Católica de Chile, Santiago, Chile; ^2^Millennium Institute for Research in Depression and Personality (MIDAP), Depression and Suicidality Research Group, Santiago, Chile; ^3^Independent researcher, Avenida José Manso de Velasco 6968, Santiago, Chile; ^4^Independent researcher, Isla Darwin 8726, Santiago, Chile; ^5^School of Nursery, Universidad San Sebastián, Santiago, Chile; ^6^School of Psychology, Universidad Diego Portales, Santiago, Chile

**Keywords:** suicide, affective disorders, artificial intelligence, risk factors, protective factors

## Abstract

**Aim:**

In efforts to develop reliable methods to detect the likelihood of impending suicidal behaviors, we have proposed the following.

**Objective:**

To gain a deeper understanding of the state of suicide risk by determining the combination of variables that distinguishes between groups with and without suicide risk.

**Method:**

A study involving 707 patients consulting for mental health issues in three health centers in Greater Santiago, Chile. Using 345 variables, an analysis was carried out with artificial intelligence tools, Cross Industry Standard Process for Data Mining processes, and decision tree techniques. The basic algorithm was top-down, and the most suitable division produced by the tree was selected by using the lowest Gini index as a criterion and by looping it until the condition of belonging to the group with suicidal behavior was fulfilled.

**Results:**

Four trees distinguishing the groups were obtained, of which the elements of one were analyzed in greater detail, since this tree included both clinical and personality variables. This specific tree consists of six nodes without suicide risk and eight nodes with suicide risk (tree decision 01, accuracy 0.674, precision 0.652, recall 0.678, specificity 0.670, *F* measure 0.665, receiver operating characteristic (ROC) area under the curve (AUC) 73.35%; tree decision 02, accuracy 0.669, precision 0.642, recall 0.694, specificity 0.647, *F* measure 0.667, ROC AUC 68.91%; tree decision 03, accuracy 0.681, precision 0.675, recall 0.638, specificity 0.721, *F* measure, 0.656, ROC AUC 65.86%; tree decision 04, accuracy 0.714, precision 0.734, recall 0.628, specificity 0.792, *F* measure 0.677, ROC AUC 58.85%).

**Conclusion:**

This study defines the interactions among a group of variables associated with suicidal ideation and behavior. By using these variables, it may be possible to create a quick and easy-to-use tool. As such, psychotherapeutic interventions could be designed to mitigate the impact of these variables on the emotional state of individuals, thereby reducing eventual risk of suicide. Such interventions may reinforce psychological well-being, feelings of self-worth, and reasons for living, for each individual in certain groups of patients.

## Introduction

Suicide is the most feared consequence of mental illness. While there are significant differences between the rates of suicide in many countries, suicide ranks among the top 15 causes of death around the world. Moreover, for every suicide, there are 20 to 30 people who make a suicide attempt (1).

Chile has a suicide rate of 11 for every 100,000 inhabitants (2013). This translates into 6 deaths per day and another 20 who unsuccessfully attempt suicide. This figure is equivalent to the number of deaths in vehicle accidents (2). The situation becomes even more alarming among young Chileans, with a 2009 suicide rate of 7 per 100,000 youths from the ages of 10–19 years, which is expected to increase to 12 per 100,000 inhabitants by the year 2020 (3).

For many years, motivated by the magnitude of this problem, national health authorities and international organizations such as the WHO (1) have explored different strategies to decrease the incidence of this behavior. Like any other important task, in this case too, one must begin by understanding the nature of this behavior.

There is a long tradition of research aimed at shedding light on the distinctive characteristics of suicidal behavior. Without a doubt, when it comes to clinical work with psychiatric patients, one of the greatest difficulties is assessing the short-term suicide risk of subjects who exhibit risk factors, as is the case for most psychiatric patients (4, 5). This limitation is in contrast with the precision of the epidemiological information that can be obtained. The rate of suicide can now be estimated in countries that keep up-to-date epidemiological records. However, we are not yet able to predict—and therefore prevent—suicide in patients who present the clinical features frequently associated with this behavior. Those who suffer from a mood disorder have a risk of suicide of around 7% throughout their lives. Moreover, while a suicide attempt is one of the factors more strongly associated with suicide, most of those who attempt it will die of a different cause (6). On the other hand, approximately 50% of those who do commit suicide never attempt it before.

The above implies that the indicators commonly associated with this behavior have poor short-term predictive value, since they are not particularly specific and are highly sensitive. There is agreement that the complexity of suicide might be attributed to the “multifactorial” nature of this complex behavior. Understanding it as “multifactorial” is to see it as a dynamic process determined by a set of factors with different properties and weights, interacting simultaneously (7, 8). The models to understand suicidal behavior usually incorporate risk factors into a process—a sequence of stages—that ultimately converge in an individual. Those who elaborate the idea of ending their lives are individuals who suffer for different reasons and, at some moment, cannot find a way to solve them. In the end, it is the individual who chooses to develop this behavior, which emerges as a response to the strain produced by the events that have caused hurt and are experienced as unbearable. This is the period that some authors highlight as the moment in which an individual has “self-destructive or suicidal thoughts” (9) and carries them out in the hope of ending his or her suffering.

Aside from acknowledging that it exists, our knowledge of the moment that precedes any suicide or suicide attempt is very limited (9–11). This serious limitation in our understanding of suicidal behavior has led many authors to question the value of suicide risk assessments in psychiatric patients (4) and to warn of the ineffectiveness of tools that aim at describing a clinical state of “imminent” suicide risk. For some, the use of risk scales or indicators to assess immediate risk should be considered not only pointless but also “dangerous,” given the high probability of incorrectly assessing the risk (12, 13). To predict and therefore prevent suicidal behavior in those suffering from mood disorders, it is necessary to study in greater detail how these factors contribute to the “at risk” condition. We understand that the multifactorial nature of suicidal behavior is also dynamic and discontinuous, which is why we have attempted to identify aspects of the interactions between clinical and personality factors that co-occur when an individual chooses to end their life. The aim of this study is to deepen our understanding of this period of great vulnerability, which precedes all suicidal behavior. We are focused on developing more reliable methods to detect the likelihood of impending suicidal behaviors, which could constitute an important step in suicide prevention science nowadays (14).

A deeper understanding of this process may help us to pinpoint the facts, factors, or circumstances that could be changed in order to bring a person out of the risk zone. Our strategy is to describe the patterns that emerge from data structures by using a vast number of variables, without any prior hypothesis. We agree with Oquendo and her colleagues, who suggested that “…Machine Learning ‘observes’ the data and ‘learns’ from it to build an understanding and uncover previously unexpected associations. In this way, this computational approach allows exploration of data to identify patterns and structures not suspected *a priori*, and thus can lead to the generation of new hypotheses…” [Ref. (15), p. 957].

By using mathematical supervised learning mechanisms, this method may enable us to establish decision rules that can recognize a temporary state of acute psychiatric discomfort that occurs before suicidal behavior (16, 17). This multifactorial behavior could be seen as a group of rules for factor interaction, each with their own features within the group. Having previously attempted suicide or being seriously depressed are risk factors for individuals, when they are part of a constellation of variables that contribute to the aforementioned state of risk. While it is highly possible that the configuration of factors may be individual (given the complex human uniqueness of psychological states), it is important to establish if there is a certain configuration that a particular group of subjects may have in common. Over recent years, data mining (DM) techniques have begun to be studied with the aim of facilitating decision-making processes in medicine (18). DM has already been used for a variety of purposes: the automated extraction and processing of emergency consultations to improve estimations of annual visits (19), the identification of adverse reactions to medication using electronic records (20), among others. The multicausal nature of psychological illnesses has led us to believe that DM could be particularly useful in studying the interaction of variables associated with illnesses or some of their manifestations, as in the case of suicide. The few studies that have been carried out with this new methodology suggest that it may be of great utility. For example, DM has been used in existing data on electronic files to estimate suicide risk (21), to establish cases of murder–suicide in the National reporting system for violent deaths (22), and to track suicide risk by following Twitter messages (23). In each of these cases, DM has shown to be an effective strategy for approaching the analysis of large quantities of data with no prior hypotheses, with the aim of identifying variables, or groups of variables that may better characterize groups of patients. Recently, our suicide research team has used a variety of DM techniques to extract variable that allow us to place patients consulting for major depression in what we call the “suicide risk zone” (24). From 345 variables initially gathered from 6 clinical and personality assessment tools, 22 variables were drawn and grouped into an assessment tool. These 22 variables were considered to define the aforementioned suicide risk zone and will be evaluated in a follow-up study with patients in therapy for suicidal behavior. The usefulness of DM for medical decisions is in the early stages of being proven and its applications and limitations are yet to be defined.

## Patients and Methods

### Participants

The sample was composed of 707 mental health patients, ages 14–85 (adolescents, young adults, adults, and seniors) using a consecutive, purposive sampling strategy. The patients were selected according to availability and in consecutive order from throughout the period defined for selecting the sample for this project (June 2010 to December 2014). These patients were undergoing treatment as usual, which, in the case of hospitalized patients, consists of crisis intervention with psychiatric, psychological, and occupational therapy approaches. For outpatients, treatment consisted of psychiatric and psychological approaches. This paper is not an intervention, but rather a cross-sectional evaluation of a specific moment. Table 1 presents the sociodemographic characteristics of the participants. Mood disorders and age distribution are shown in Tables 2 and 3. They were classified into two groups: (1) a group with suicidal behavior as indicated by consultations relating to a suicide attempt or presenting suicidal ideation in the preceding years and (2) a group without suicidal behavior, who attended mental health consultations without having made a suicide attempt or having presented recent suicidal ideation. Psychiatric diagnoses were made in collaboration with the treating teams, according to the diagnostic criteria set out in the Diagnostic and Statistical Manual of Mental Disorders, 4th Edition published by the American Psychiatric Association (25).

**Table 1:** **Sociodemographic characteristics of the sample, differences between groups**.

Variable	Total	Group without current suicidal behavior	Group with suicidal behavior	Test
*N*	707	358	349	
Mean	39.68%	42.16%	37.16%	*t* = −4.4993
				df = 704
				*p* = 7.975e−06**
SD	14.849	14.459	14.843	

**Sex *n*/%**				χ^2^ = 0.029053
Female	564	287	277	df = 1
	79.774	80.168	79.370	
Male *n*	143	71	72	*p* = 0.8647
	20.226	19.832	20.630	

**Marital status *n*/%**				χ^2^ = 13.12
Married	259	148	110	df = 3
	36.634	41.341	31.519	
Unmarried	33	19	13	*p* = 0.004378**
	4.668	5.31	3.72	
Single	295	127	169	
	41.726	35.475	48.424	
Divorced or widower	120	64	57	
	16.973	17.877	16.332	

**With children *n*/%**	454	248	206	χ^2^ = 8.0851
	64.215	69.274	59.0258	df = 1
				*p* = 0.004463**

**Completed educational level *n*/%**				χ^2^ = 4.0694
With higher education	333	154	179	df = 1
	47.100	43.017	51.289	*p* = 0.04367*
Without higher education	374	204	170	
	52.900	56.983	48.711	

**Occupation *n*/%**				χ^2^ = 25.91
Employed	375	221	154	df = 3
	53.041	61.732	44.126	
Student	157	56	101	*p* = 9.92e−06**
	22.207	15.642	28.940	
Unemployed	42	20	22	
	5.941	5.587	6.304	
Housewife	133	61	72	
	18.812	17.039	20.630	

*p < 0.05.

**p < 0.001.

**Table 2:** **Mood disorders distribution, differences between groups**.

Variable *N* (%)	Total	Group without current suicidal behavior	Group with suicidal behavior	Test
Major depressive disorder	311	106 (34.08%)	205 (65.93%)	χ^2^ = 67.75
Bipolar disorder	112	62 (55.36%)	50 (44.64%)	
Moderate depressive disorder	53	30 (56.60%)	23 (43.40%)	
Mild depressive disorder	13		1 (6.69%)	df = 8
Anxiety disorder	74	12 (92.31%)	22 (29.73%)	
Mixed episode	14	52 (70.27%)	2 (14.29%)	
Adjustment disorder	73	12 (85.71%)	27 (36.99%)	*p* = 1.37e−11*
Dysthymia	8	45 (63.01%)	3 (37.50%)	
Others disorders	29	5 (62.50%)	14 (48.28%)	
		15 (51.72%)		
	(*n* = 687)	(*n* = 340)	(*n* = 347)	

*p < 0.001.

**Table 3:** **Age distribution, differences between groups**.

Variable *N* (%)	Total	Group without current suicidal behavior	Group with suicidal behavior	Test
14–19 years	80	25 (31.25%)	55 (68.75%)	χ^2^ = 28.82
20–29 years	130	57 (43.85%)	73 (56.15%)	
30–39 years	135	66 (48.89%)	69 (51.11%)	df = 5
40–49 years	142	85 (59.86%)	57 (40.14%)	
50–59 years	156	81 (51.92%)	75 (48.08%)	*p* = 2.51e−05*
60 years and more	63	44 (69.84%)	19 (30.16%)	
	(*n* = 706)	(*n* = 358)	(*n* = 348)	

*p < 0.001.

Voluntary participation was requested from subjects along with informed consent/assent. This document was designed using the ethical criteria for research using humans (26). The protocol was approved by the institutional ethics committees of the School of Medicine at Universidad Católica de Chile and the Sótero del Río Hospital. The sample comprised patients consulting for mental health issues, between the ages of 14 and 83, at walk-in and inpatient facilities at three health-care centers, serving different socioeconomic levels in the Greater Santiago, Chile. Participant recruitment and data collection were carried out between June 2010 and December 2014.

The inclusion criteria covered subjects consulting for mental health issues, over the age of 14 and into advanced adulthood, of both sexes, who were able to distinguish reality from fantasy and who made informed consent/assent and demonstrated their availability to participate in the study, and who were in a cognitive and emotional state that allowed them to answer the assessment questions. The exclusion criteria, for methodological reasons and in order to control the diagnostic variable, covered subjects consulting for addiction, eating disorders, psychotic disorders, or cognitive disorders. The exclusion of these pathologies was decided in view of the methodological aim of focusing the analysis on mood disorders, even though the pathologies excluded from this study are also highly linked with suicide risk (2, 27, 28). In addition, those who chose not to participate in the study or those who later withdrew having initially accepted were not included.

Using prior qualitative–quantitative studies, results were obtained with regard to selecting the relevant clinical variables and personalities that protect from suicide risk or place someone at risk. These include psychological distress resulting in dysfunctionality, a dysfunctional experience and expression of aggression, reasons that prevent suicidal behavior, destructive depressive experiences, and satisfaction with family functioning (29–31). These findings led to the selection of the instruments detailed below, which introduced 345 study variables overall:

### Tools

The validated Spanish version of the Outcome Questionnaire (OQ-45.2) (32, 33) assesses how a person has been feeling over the preceding few days with regard to (a) anxious and depressive symptomatology, (b) interpersonal relationships, and (c) feelings of adaptation to social roles (family roles, employment, and leisure). The internal consistency of the tool validated for Chile shows a Chronbach’s alpha of α = 0.930 for the overall scale; α = 0.910 for the anxious and depressive symptomatology subscale; α = 0.740 for the well-being/discomfort in interpersonal relationships subscale, and α = 0.710 for the feelings of adaptation to social roles subscale.

The validated Spanish version of the State Trait Anger Expression Inventory (34) assesses the experience of anger from the patient’s point of view from two perspectives: (a) state of anger and trait of anger and (b) the expression of anger in three ways: (1) loss of control, (2) overcontrol, and (3) functional control. Its internal consistency shows a Chronbach’s alpha for the study sample of α = 0.779 for the overall scale; α = 0.875 for the state of anger subscale; α = 0.809 for the trait of anger subscale; α = 0.842 for the control subscale; α = 0.603 for the subscale of suppressing anger, and α = 0.654 for the expressing anger subscale.

The validated Spanish version of the reasons for living (RFL) scale (35, 36) assesses reasons for not committing suicide from six perspectives according to the importance that the patient him or herself accords to (a) confidence in his or her ability to face difficult situations; (b) fear of death and social disapproval; (c) family responsibility; (d) concern for children; (e) a perception of an inability to commit suicide; and (f) objections of a moral nature: the internal consistency shows a Chronbach’s alpha in the sample studied of α = 0.950 for the overall scale; α = 0.956 for the confidence in one’s ability to face difficult situations subscale; α = 0.750 for the fear of death and social disapproval subscale; α = 0.821 for the family responsibility subscale; α = 0.872 for the concern for children subscale; α = 0.722 for the perception of an inability to commit suicide subscale, and α = 0.771 for the moral objections subscale. This scale has been validated for Chile by our research team and is in the process of being published.

The validated Spanish version of the Depressive Experience Questionnaire (37, 38) measures two factors that relate to vulnerability and are associated with depression: (a) self-criticism and (b) dependence. There is a third factor, which is thought to protect subjects from suicide risk and relates to (c) self-efficacy. The internal consistency shows a Chronbach’s alpha for the sample studied of α = 0.844 for the overall scale; α = 0.60 for the dependency subscale; α = 0.79 for the self-criticism subscale, and α = 0.69 for the self-efficacy subscale.

The validated Spanish version of the Family APGAR (39, 40) measures satisfaction with family functioning through a general assessment of five aspects of the respondent’s family life: (a) adaptability; (b) participation; (c) growth gradient; (d) affection; and (e) resolution. The internal consistency of this tool validated for Chile shows an α = 0.79.

A questionnaire containing descriptive information about the patient with regards to their diagnosis and sociodemographic–clinical background has also been used.

### Data Collection Procedure

A deliberate sample was chosen based on the availability of patients in the various services, who were then evaluated consecutively. The aforementioned inclusion criteria were taken into account, and the clinical diagnoses were made together with the treating teams. The study was explained, and voluntary participation was requested. Potential subjects were asked to sign the informed consent/assent form, and they were then asked to respond to the questions included in the various tools, guided by specially trained assessors. The informed consent/assent forms were approved in advance by the institutional ethics committees. In the event that participants were minors, the informed consent and signature of the guardian or caregiving father/mother was also requested in addition to the assent and signature. The aim of the study and the methodology were explained as well as the fact that it was unpaid and the costs, risks, voluntary nature of participation, their right to withdraw from the study, and information confidentiality. The authorization of the treating physician was also sought for the participation of patients and any potential deterioration in mental state during the research was to be noted. No incidents were recorded during this study. Participants were also offered the opportunity to further inquire about the study by contacting the head researcher (SM).

### Data Analysis Procedure

These were undertaken in multidisciplinary collaboration between clinician-researchers in mental health and mathematical analysts.

Differences were identified between the two defined groups with suicidal behavior (being treated for suicide attempts or suicidal ideation) and without suicidal behavior (receiving treatment for other reasons without suicide attempts or suicidal ideation). The initial database with its raw data comprised 707 assessments and 345 variables, corresponding to the questions from the assessment tools and the sociodemographic details of the recruited patients.

Classifications were made based on the collected demographic data with regard to the characteristics of the patients, such as the mental health center, gender, marital status, age, level of education, and whether or not they had children.

Analysis was carried out using artificial intelligence and DM tools, which enabled the different variables to be interpreted in a systematic and automated way (41). Given the nature of the problem, supervised learning models were selected from among the techniques in this field to calibrate the algorithms using a series of examples, known as training data, which already have a defined set of variables and are already linked to an answer relating to that set of variables (42). This was used to generate a model that could predict new cases.

The analyses were carried out using two techniques: (1) the Cross Industry Standard Process for Data Mining (CRISP-DM) methodology (43) and decision and (2) decision tree analysis.

Cross Industry Standard Process for Data Mining methodology is widely used to resolve DM problems across a range of industries and has come to be seen as the standard methodology (44). It is usually applied in multidisciplinary contexts when it is necessary to understand both the problem and the analysis technique in all of their detail. It comprises six phases that enable joint collaboration, namely business understanding, data understanding and preparation, modeling, evaluation, and deployment.

Data mining decision trees are an effective predictive model used in artificial intelligence for analyzing the interaction between a large number of explanatory variables including dichotomous and continuous variables, thereby allowing for easy interpretation and clinical application of the results (45). This analytical tool was used to generate logical diagrams categorizing certain conditions for belonging to a configuration of personal and clinical suicide risk variables. It indicates a route to be followed depending on the value that the variable reaches, and it is represented in the form of a tree, whose branches split depending on the values attained by the variables and end in an action: belonging in the risk situation or not.

The analytical process built a series of logical rules (nodes) in order to divide the data based on the group of attributes for each entry (46). The basic algorithm used was a top-down algorithm that looks for different rules to define groups of interest and aims to increase the branching of the first decision tree obtained. In the results that we will present, after showing the decision trees obtained, we have chosen to enter into a deeper explanation of decision tree no 3 for illustrative purposes only. We felt this to be the most suitable example for explaining the model, due to the sufficient quantity of variables and the depth of the clinical aspects from the assessment (45).

Initially, the algorithm assessed all available data and all of the possible divisions that could be carried out. Subsequently, the most appropriate division generated by the tree was chosen according to the Gini index criterion (45).

This index measures the level of purity of a particular node. The aim is to keep the sum of the Gini indices of all of the nodes to a minimum, thereby reducing the probability that a final node will have different types of records (47).

The data were divided into two subgroups and were then looped until any ultimate condition was reached (either belonging to the group with suicidal behavior or belonging to the group without suicidal behavior). The Gini index was calculated for each possible division using, in this case, the two options available for the objective variable. With P1 (shown in the supplied image of decision trees) taken as the proportion of individuals who, given the proposed division would belong to class 1 of the objective variable (without suicidal behavior), and P2 taken as the proportion of individuals belonging to class 2 (with suicidal behavior), the Gini index of the proposed division was calculated as follows:

$$\text{Gini index}=1-{\left(p_{0}\right)}^{2}-{\left(p_{1}\right)}^{2}.$$

Gini index =1−probability of obtaining records of the same class.

The criterion was to choose the division that had the lowest Gini index (47). Within the classification conditions, it was established that at a particular node (decision point) all of the records belonged to a single class, the number of observations were below a set amount beyond which division was not attempted. The number of observations belonging to the lowest class in a node was less than a set number or that of the reduction in the overall fit.

This criterion ensures that, for each final node, only records belonging to a single class are obtained and that the number of observations remains below a set number. As a result, no attempt is made to create a division; or to ensure that the number of observations belongs to a lower class or that the overall lack of a node is less than a certain number (48).

The methodological decision was taken to prune the decision trees, removing certain nodes without reducing the overall fit of the model. In order to achieve this, a number of crossed repetitions were carried out. The aim of this was to obtain a series of models that would supply the logic for sequential rules to determine when a certain individual belongs to a suicide risk configuration.

For a model to have good predictive ability, its sensitivity should ideally be high regardless of the cutoff point selected and the FPR ratio. The larger generated “area under the curve” (AUC) is, the greater the predictive abilities of the model (49).

In addition to the ratios described, a receiver operating characteristic (ROC) curve was used to graphically assess the discriminatory abilities of the model that classified observations into two groups (50). Different cutoff points were analyzed as part of this process. Based on these cutoff points, the sensitivity and specificity of the model were calculated. Afterward, “sensitivity” (or TPR) on the “*y* axis” was represented on a graph against “1-specificity” (or FPR) on the “*x* axis.” For a model with good predictive ability, its sensitivity should ideally always be high, regardless of the cutoff point selected and the FPR ratio. The larger the generated “Area Under the Curve” is, the greater the predictive abilities of the model.

The analysis issue was approached from two perspectives in order to obtain a wider range of rules to contribute to the aims of the study. Each approach that was developed included two stages, and a decision tree was obtained from each. Finally, the models obtained were assessed using cross-validation.

The first approach consisted of using all of the available variables, i.e., both those relating to the sociodemographic profiles of participants and those obtained through the application of the various tools. The second approach only used the variables that resulted from the assessment tools used.

As a first step, each approach was developed with the Gini index as a criterion for dividing the data. Subsequently, during the second phase, the tree was pruned, minimizing its predictive error when faced with a cross-validation technique. This tool trained the analysis for 80% of the data and assessed the rules obtained using the remaining 20% of the data. The procedure was repeated five times in order to use all of the available information to train the analysis undertaken.

#### Performance Measurements

Sensitivity measurement was defined as the ability of the model to correctly detect which patients were in a suicide-risk configuration. The specificity measurement was the model’s ability to correctly detect which patients were not configured as part of the group with suicidal behavior. The accuracy indicator was the ability to correctly classify the group with suicidal behavior. The precision indicator was the proportion of people with suicidal behavior that was correctly classified. In order to calculate the metrics above, a cutoff probability of 0.5 was set to determine whether an individual would be classified as “belonging to a suicide risk configuration. ”.

The progressive development of the results obtained from the two lines of analysis is presented below.

The sample comprised 707 consultants, of which 97% were diagnosed with mood disorders (DSM IV-R): with suicidal behavior group—current suicide ideation—current suicide attempt—(*n* = 349) and without suicidal behavior group (*n* = 358). The average age was 39.68 ± 14.85 years, with a range of 14–83 years; 79.77% (*n* = 564) were women, and 20.23% (*n* = 143) were men; 36.63% (*n* = 259) were married, and 41.73% (*n* = 295) were single; 47.10% (*n* = 333) had completed higher education, and 53.04% (*n* = 375) were employed (Table 1). Mood disorders and age distribution are shown in Tables 2 and 3.

#### Results of the Decision Trees

**Figure 1:**
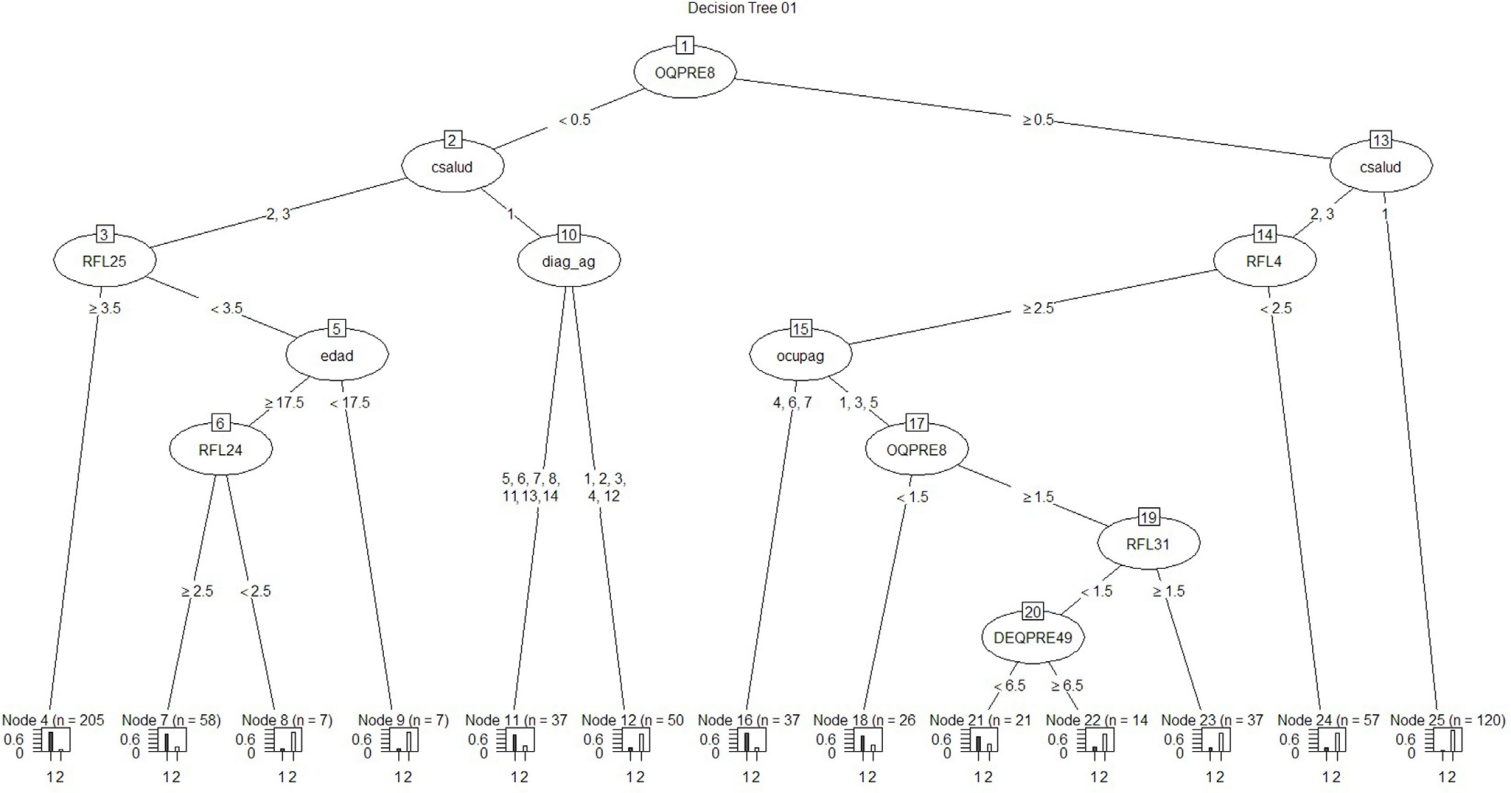
**Decision tree 01**.

**Figure 2:**
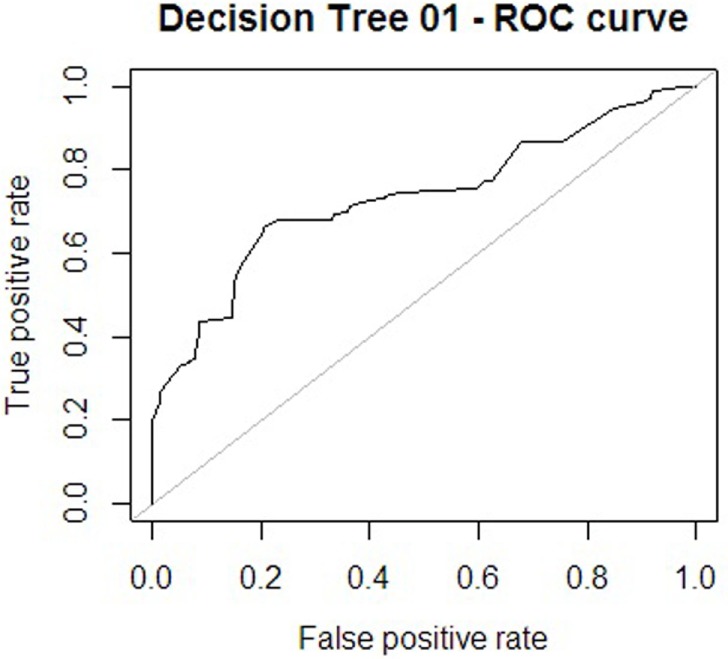
**Receiver operating characteristic (ROC) curve 1**.

**Figure 3:**
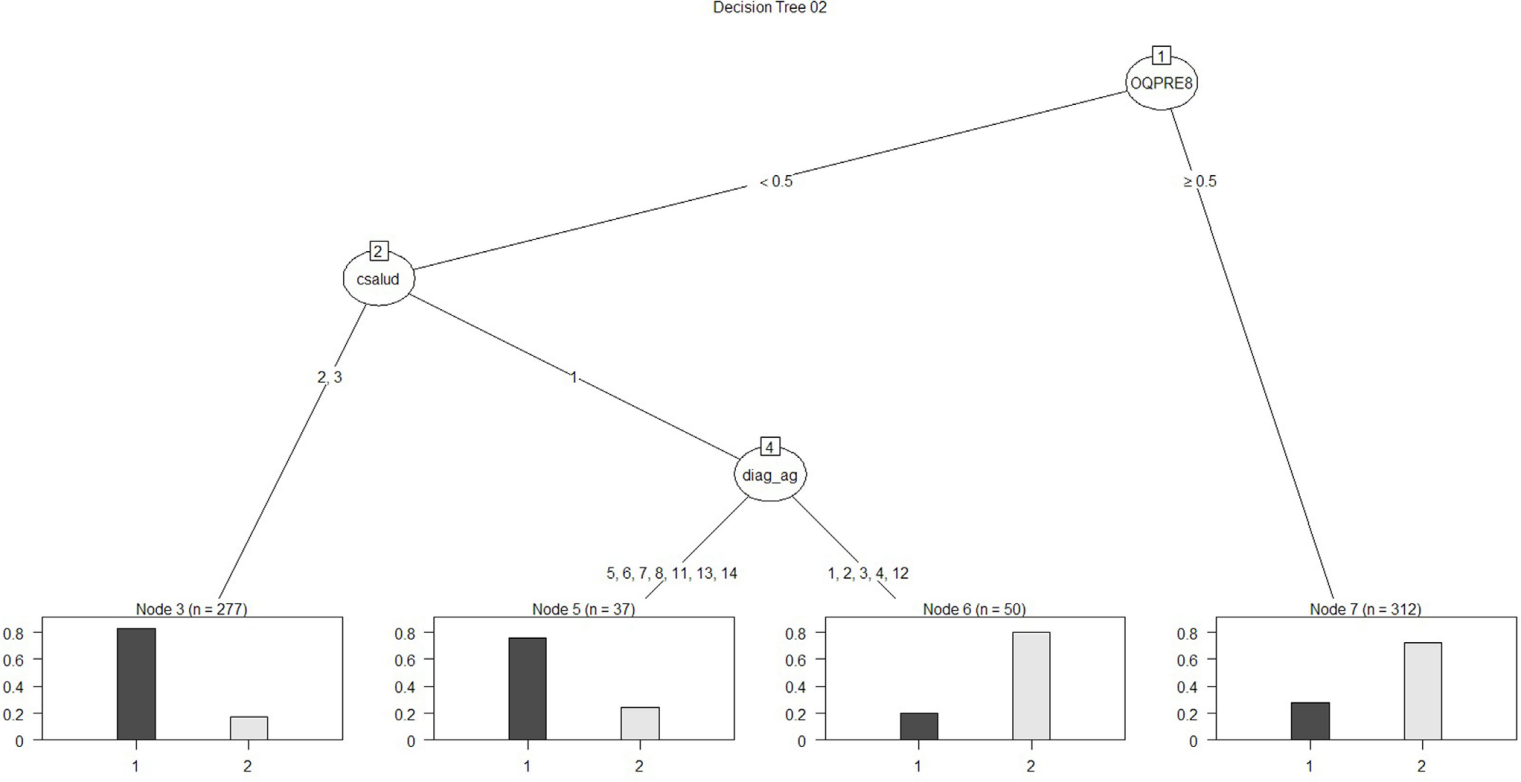
**Decision tree 02**.

**Figure 4:**
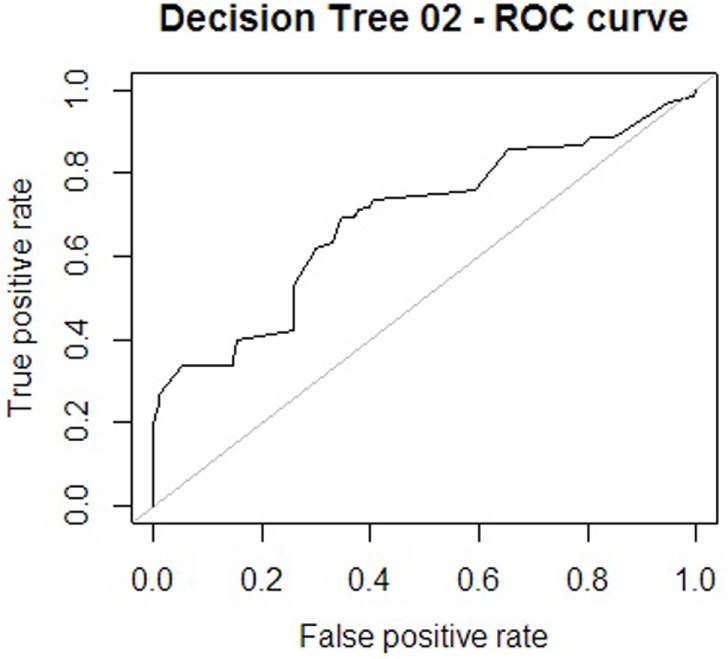
**Receiver operating characteristic (ROC) curve 2**.

Regarding the first approach mentioned (the patient’s sociodemographic profile and tool variables), the first model obtained is shown in Figure 1. It has 12 nodes, 7 of which correspond to tool variables. Five of these correspond to variables associated with the sociodemographic profile of the consulting patient. Figure 2 presents the ROC curve obtained for this model. The AUC shows a 73.35% of predictive abilities for the model. From this same approach, the model obtained through optimal pruning is shown in Figure 3. The ROC curve for this model is shown in Figure 4, and the AUC shows a 68.91% of predictive abilities of the model.

**Figure 5:**
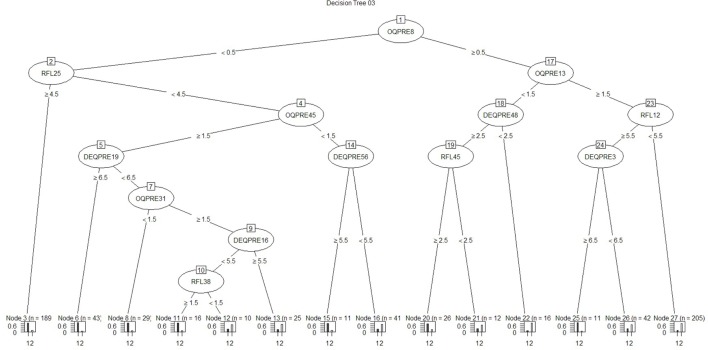
**Decision tree 03**.

**Figure 6:**
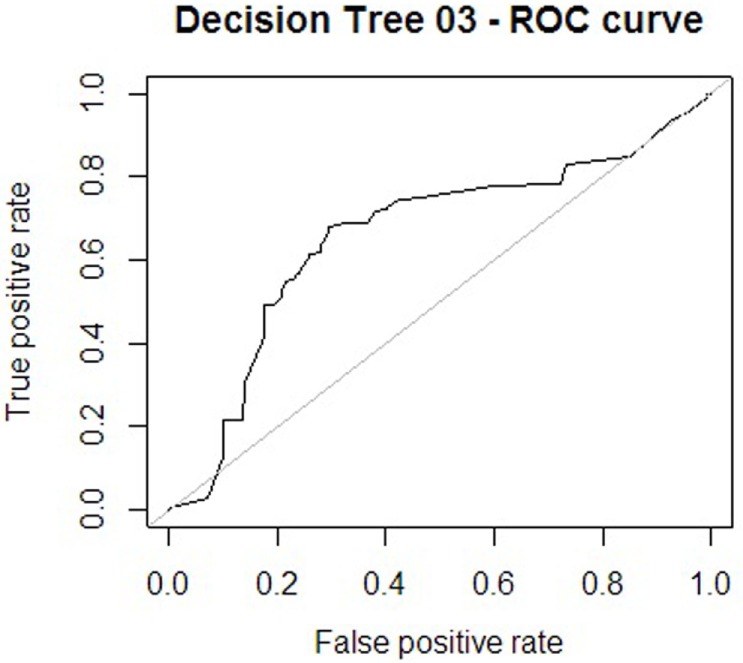
**Receiver operating characteristic (ROC) curve 3**.

**Figure 7:**
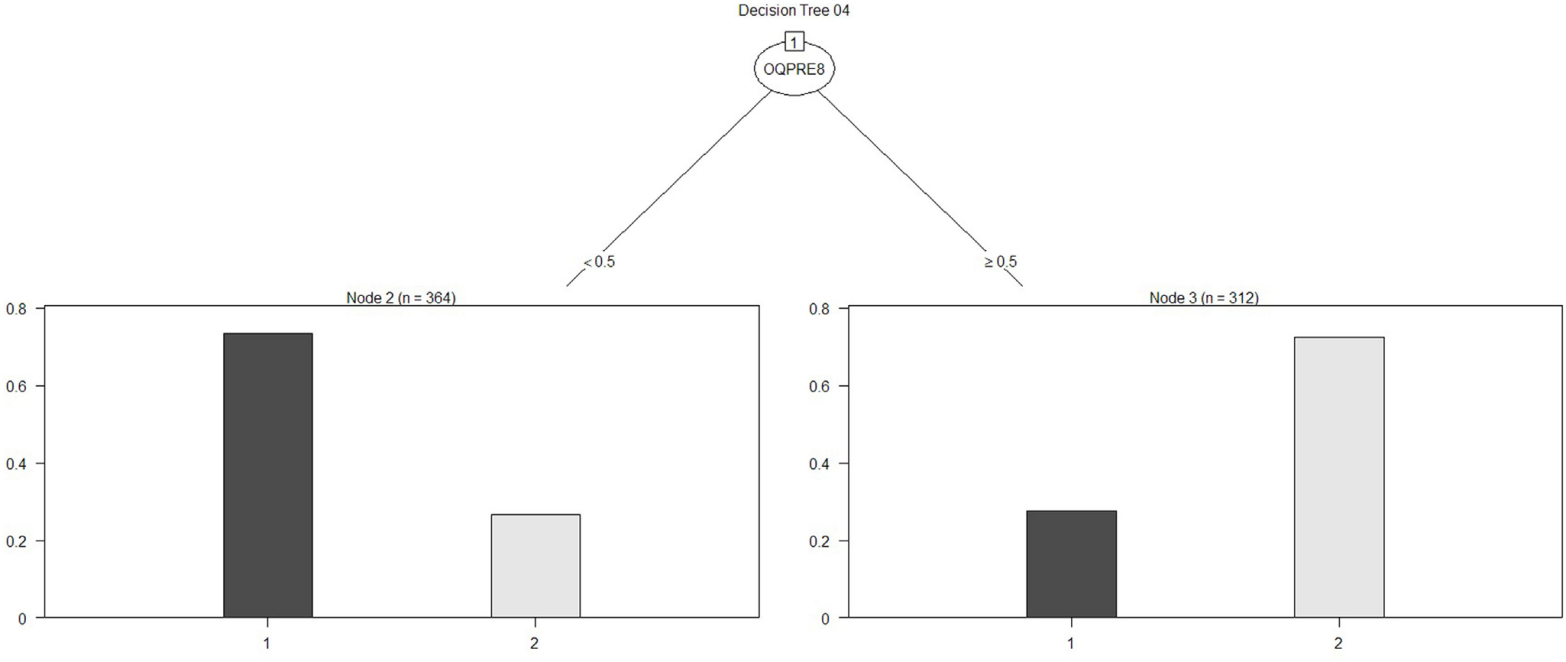
**Decision tree 04**.

**Figure 8:**
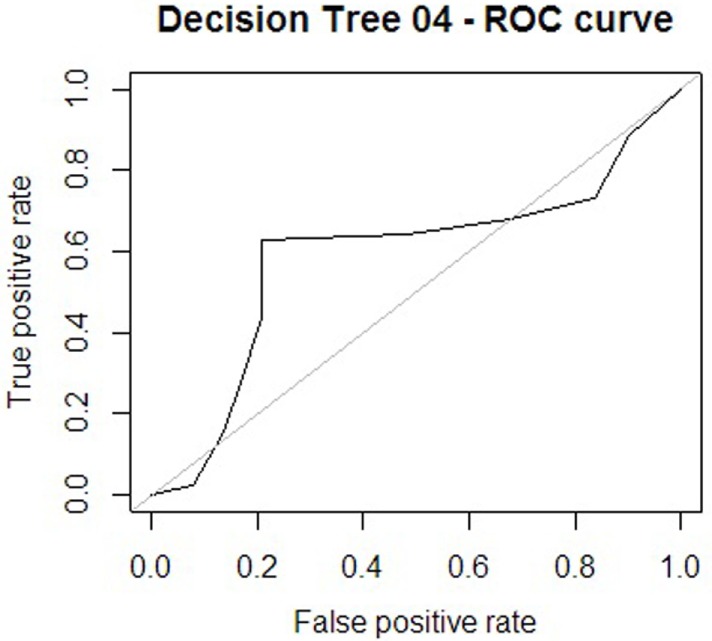
**Receiver operating characteristic (ROC) curve 4**.

For the other analytical approach (assessment tool variables), the first model obtained is presented in Figure 5, and its ROC can be seen in Figure 6. The AUC shows a 65.86% of predictive abilities for the model. Finally, the model that resulted after the aforementioned model was subjected to optimal pruning is shown in Figure 7. The AUC ROC shows a 58.85% of predictive abilities for the model, and its curve is presented in Figure 8.

The performance measurements used to compare the different models generated are presented in Table 4.

**Table 4:** **Performance measures models**.

Metric	Tree decision 01	Tree decision 02	Tree decision 03	Tree decision 04
Accuracy	0.674	0.669	0.681	0.714
Precision	0.652	0.642	0.675	0.734
Recall	0.678	0.694	0.638	0.628
Specificity	0.670	0.647	0.721	0.792
F measure	0.665	0.667	0.656	0.677

Of the four decision trees obtained using the looping process, the decision was taken to further investigate the substantive aspects of decision tree no 3. This model was preferred for illustrative purposes. While decision tree no 4 ranked higher in terms of both accuracy and *F* measure, and for recall decision tree no 3 performed less well than decision trees no 1 and no 2, it nonetheless appeared to be the example most suited to explaining the model with regard to the sufficient number of variables and the depth of the clinical aspects from the assessments, in order to identify the risk zone in which the patients found themselves. This tree also performed well transversally across the different metrics compared. The decision tree continues to show the flow of responses as a trajectory of psychological variables that constitute the current situation of suicide risk (or otherwise) as described below in Table 5.

**Table 5:** **Detail of variables decision tree**.

	Condition	Item	Answer
**Node 3 (NSR)**			
OQ 8	<0.5	I have thoughts of ending my life	Never
RFL 25	≥4.5	I am too stable to kill myself	Quite important and extremely important
**Node 6 (NSR)**			
OQ 8	<0.5	I have thoughts of ending my life	Never
RFL 25	<4.5	I am too stable to kill myself	Not at all important, quite unimportant, somewhat unimportant, and somewhat important
OQ 45	≥1.5	I have headaches	Sometimes, frequently, and always
DEQ 19	≥6.5	I become frightened when I feel alone	Strongly agree
**Node 8 (NSR)**			
OQ 8	<0.5	I have thoughts of ending my life	Never
RFL 25	<4.5	I am too stable to kill myself	Not at all important, quite unimportant, somewhat unimportant, and somewhat important
OQ 45	≥1.5	I have headaches	Sometimes, frequently, and always
DEQ 19	<6.5	I become frightened when I feel alone	Strongly disagree, disagree, fairly agree, agree, and strongly agree
OQ 31	<1.5	I am satisfied with my life	Frequently and always
**Node 11 (NSR)**			
OQ 8	<0.5	I have thoughts of ending my life	Never
RFL 25	<4.5	I am too stable to kill myself	Not at all important, quite unimportant, somewhat unimportant, and somewhat important
OQ 45	≥1.5	I have headaches	Sometimes, frequently, and always
DEQ 19	<6.5	I become frightened when I feel alone	Strongly disagree, disagree, fairly agree, agree, and strongly agree
OQ 31	≥1.5	I am satisfied with my life	Sometimes, rarely, and never
DEQ 16	<5.5	There are times when I feel “empty” inside	Strongly disagree, disagree, fairly agree, and agree
RFL 38	≥1.5	I am afraid of the actual “act” of killing myself	Quite unimportant, somewhat unimportant, somewhat important, quite important, and extremely important
**Node 12 (SR)**			
OQ 8	<0.5	I have thoughts of ending my life	Never
RFL 25	<4.5	I am too stable to kill myself	Not at all important, quite unimportant, somewhat unimportant, and somewhat important
OQ 45	≥1.5	I have headaches	Sometimes, frequently, and always
DEQ 19	<6.5	I become frightened when I feel alone	Strongly disagree, disagree, fairly agree, agree, and strongly agree
OQ 31	≥1.5	I am satisfied with my life	Sometimes, rarely, and never
DEQ 16	<5.5	There are times when I feel “empty” inside	Strongly disagree, disagree, fairly agree, and agree
RFL 38	<1.5	I am afraid of the actual “act” of killing myself	Quite unimportant
**Node 13 (SR)**			
OQ 8	<0.5	I have thoughts of ending my life	Never
RFL 25	<4.5	I am too stable to kill myself	Not at all important, quite unimportant, somewhat unimportant, and somewhat important
OQ 45	≥1.5	I have headaches	Sometimes, frequently, and always
DEQ 19	<6.5	I become frightened when I feel alone	Strongly disagree, disagree, fairly agree, agree, and strongly agree
OQ 31	≥1.5	I am satisfied with my life	Sometimes, rarely, and never
DEQ 16	>5.5	There are times when I feel “empty” inside	Strongly agree
**Node 15 (NSR)**			
OQ 8	<0.5	I have thoughts of ending my life	Never
RFL 25	<4.5	I am too stable to kill myself	Not at all important, quite unimportant, somewhat unimportant, and somewhat important
OQ 45	<1.5	I have headaches	Never and rarely
DEQ 56	≥5.5	In my relationships with others, I am very concerned about what they can give to me	Strongly agree
**Node 16 (SR)**			
OQ 8	<0.5	I have thoughts of ending my life	Never
RFL 25	<4.5	I am too stable to kill myself	Not at all important, quite unimportant, somewhat unimportant, and somewhat important
OQ 45	<1.5	I have headaches	Never and rarely
DEQ 56	<5.5	In my relationships with others, I am very concerned about what they can give to me	Strongly disagree, disagree, fairly agree, agree, and strongly agree
**Node 20 (NSR)**			
OQ 8	≥0.5	I have thoughts of ending my life	Rarely, sometimes, frequently, and always
OQ 13	<1.5	I am a happy person	Frequently and always
DEQ 48	≥2.5	I feel good about myself whether I succeed or fail	Disagree, fairly agree, agree, and strongly agree
RFL 45	≥2.5	I see no reason to hurry death along	Somewhat unimportant, somewhat important, quite important, and extremely important
**Node 21 (SR)**			
OQ 8	≥0.5	I have thoughts of ending my life	Rarely, sometimes, frequently, and always
OQ 13	<1.5	I am a happy person	Frequently and always
DEQ 48	≥2.5	I feel good about myself whether I succeed or fail	Disagree, fairly agree, agree, and strongly agree
RFL 45	<2.5	I see no reason to hurry death along	Not important and quite unimportant
**Node 22 (SR)**			
OQ 8	≥0.5	I have thoughts of ending my life	Rarely, sometimes, frequently, and always
OQ 13	<1.5	I am a happy person	Frequently and always
DEQ 48	<2.5	I feel good about myself whether I succeed or fail	Strongly disagree
**Node 25 (NSR)**			
OQ 8	≥0.5	I have thoughts of ending my life	Rarely, sometimes, frequently, and always
OQ 13	≥1.5	I am a happy person	Never, rarely, and sometimes
RFL 12	≥5.5	Live is all we have and is better than nothing	Extremely important
DEQ 3	≥6.5	I tend to be satisfied with my current plans and goals, rather than striving for higher goals	Strongly agree
**Node 26 (SR)**			
OQ 8	≥0.5	I have thoughts of ending my life	Rarely, sometimes, frequently, and always
OQ 13	≥1.5	I am a happy person	Never, rarely, and sometimes
RFL 12	≥5.5	Live is all we have and is better than nothing	Extremely important
DEQ 3	<6.5	I tend to be satisfied with my current plans and goals, rather than striving for higher goals	Strongly agree, agree, fairly agree, disagree, strongly disagree, and total disagree
**Node 27 (SR)**			
OQ8	≥0.5	I have thoughts of ending my life	Rarely, sometimes, frequently, and always
OQ13	≥1.5	I am a happy person	Never, rarely, and sometimes
RFL 12	<5.5	Live is all we have and is better than nothing	Quite unimportant, somewhat unimportant, somewhat important, and quite important

*NSR, non-suicide risk (without risk of ideation or attempt); SR, with suicide risk; RFL, reasons for living; OQ, Outcome Questionnaire; DEQ, Depressive Experience Questionnaire*.

The variables generated by decision tree no 3 are explained substantively. The decision tree demonstrates the flow of responses in a trajectory of psychological variables that constitute a current state of suicide risk (or otherwise) as described below:

**Substantive wording of answers that situate the patient in the not at risk zone (according to decision tree no 3)**.
Node 3: never having thought about taking one’s life in the last 7 days; considering that feeling too stable to commit suicide is very significant or extremely significant as a reason not to commit suicide.
Node 6: never having thought about taking one’s life in the last 7 days; considering that feeling too stable to commit suicide is insignificant, very insignificant, not very significant, or significant as a reason not to commit suicide; having experienced headaches in the past 7 days at times, frequently, and almost always; not totally agreeing with the statement “I am terrified when I feel alone”; having frequently or almost always felt satisfied with life in the last 7 days.
Node 8: never having thought about taking one’s life in the last 7 days; considering that feeling too stable to commit suicide is insignificant, very insignificant, not very significant, or significant as a reason not to commit suicide; having experienced headaches in the past 7 days at times, frequently, and almost always; not totally agreeing with the statement “I am terrified when I feel alone”; having frequently or almost always felt satisfied with life in the last 7 days.
Node 11: never having thought about taking one’s life in the last 7 days; considering that feeling too stable to commit suicide is very insignificant or not very significant as a reason not to commit suicide; having experienced headaches in the past 7 days frequently and almost always; not totally agreeing with the statement “I am terrified when I feel alone”; having at times, almost never, and never felt satisfied with life in the last 7 days; not totally agreeing with the statement “At times I feel empty inside.”
Node 15: never having thought about taking one’s life in the last 7 days; considering that feeling too stable to commit suicide is very insignificant or not very significant as a reason not to commit suicide; never having experienced headaches in the last 7 days; very much agreeing and totally agreeing with the statement “In my relationships with others I think a lot about what they can give me.”
Node 20: never having thought about taking one’s life in the last 7 days; frequently and almost always feeling like a happy person; not totally agreeing or totally disagreeing with the statement “I feel great about myself whether I succeed or fail”; considering the sentence “I don’t see any reason to bring death forward” as anywhere from not very important to extremely important.
**Substantive wording of answers that situate the patient in the not at risk zone (according to decision tree no 3)**.
Node 12: never having thought about taking one’s life in the last 7 days; considering that feeling too stable to commit suicide is insignificant, very insignificant, not very significant, or significant as a reason not to commit suicide; having experienced headaches in the past 7 days at times, frequently, and almost always; not totally agreeing with the statement “I am terrified when I feel alone”; having at times, almost never, and never felt satisfied with life in the last 7 days; totally disagreeing, very much disagreeing, slightly disagreeing, and agreeing with the statement “At times I feel empty inside”; considering that a fear of committing the act of suicide itself is very insignificant as a reason not to commit suicide.
Node 13: never having thought about taking one’s life in the last 7 days; considering that feeling too stable to commit suicide is insignificant, very insignificant, not very significant, or significant as a reason not to commit suicide; having experienced headaches in the past 7 days at times, frequently, and almost always; not totally agreeing with the statement “I am terrified when I feel alone”; having at times, almost never, and never felt satisfied with life in the last 7 days; totally agreeing and very much agreeing with the statement “At times I feel empty inside.”
Node 16: never having thought about taking one’s life in the last 7 days; considering that feeling too stable to commit suicide is insignificant, very insignificant, not very significant, or significant as a reason not to commit suicide; never or almost never having experienced headaches in the past 7 days; not totally agreeing or very much agreeing with the statement “In my relationships with others I think a lot about what they can give me.”
Node 21: almost always and frequently having thought about taking one’s life in the last 7 days; frequently and almost always feeling like a happy person in the last 7 days; agreeing, slightly agreeing, very much agreeing, or totally agreeing with the statement “I feel good about myself whether I succeed or fail”; considering not seeing any reason to bring death forward as insignificant or very insignificant as a reason not to commit suicide.
Node 22: almost never, at times, and almost always having thought about taking one’s life in the last 7 days; frequently and almost always feeling like a happy person in the last 7 days; totally agreeing or very much disagreeing with the statement “I feel good about myself whether I succeed or fail.”
Node 25: almost never, at times, frequently, and almost always having thought about taking one’s life in the last 7 days; almost never or at times feeling like a happy person in the last 7 days; feeling that the fact that this life is the only one we’ve got and it’s better than nothing is extremely significant as a reason not to commit suicide; totally agreeing with the statement “I generally feel that I am more suited to my plans and goals than trying to achieve higher objectives.”
Node 26: almost never, at times, frequently, and almost always having thought about taking one’s life in the last 7 days; almost never or at times feeling like a happy person in the last 7 days; feeling that the fact that this life is the only one we’ve got and it’s better than nothing is extremely significant as a reason not to commit suicide; very much agreeing, agreeing, slightly agreeing, disagreeing, very much disagreeing, or totally disagreeing with the statement “I generally feel that I am satisfied with to my plans and goals rather than trying to achieve higher objectives.”
Node 27: almost never, at times, frequently, and almost always having thought about taking one’s life in the last 7 days; almost never or at times feeling like a happy person in the last 7 days; feeling that the fact that this life is the only one we’ve got and it’s better than nothing is not significant, very insignificant, not very significant, significant, and very significant as a reason not to commit suicide.

These results translate into the following configuration by group. Without suicide risk: not having thought about taking one’s life, feeling good about oneself (whether one succeeds or fails), not feeling empty inside, frequently feeling like a happy person and being satisfied with life, in addition to being highly concerned with what you receive from others. With suicide risk: having thought about taking one’s life, having experienced frequent headaches, having been unsatisfied or not very satisfied with life lately, feeling empty inside at times, and not feeling like a happy person.

## Discussion

The use of decision tree techniques in artificial intelligence has allowed us to look at the state that precedes suicide. It is a process that is usually temporary, experienced as psychological discomfort, and in some cases, it feels like a psychologically unbearable moment (4, 5, 51). Using 345 variables that correspond to tools assessing subjective well-being, state, trait-expression of anger, depressive lifestyle, satisfaction with family functioning, and RFL, it was possible to generate four decision trees that can distinguish between groups with and without suicidal behavior. In this paper, we focus on one of these trees, which had 14 nodes, 6 of which related to the group without suicidal behavior and the remaining 8 corresponding to the group with suicidal behavior. These analyses have led us to see which factors are useful in detecting a person’s vulnerability and can also guide psychotherapeutic interventions that might relieve and reduce the probability of future suicide attempts.

The knowledge contributed by these findings may mark a change in clinical application; through the use of tailored psychotherapeutic interventions to strengthen factors that protect from suicide and minimize factors that make a person vulnerable, for each individual case assessed using the techniques set out in this paper. The analytical methods we have proposed are new, and there are few studies on this topic that make use of DM tools. However, some recent research studies have resulted in similar recommendations that emphasize how these techniques can be applied to clinical practice (15, 52). Regarding the specific variables that we have found in this study, there are some similarities with findings obtained using other, more traditional, statistical techniques, which confirm their significance and strongly encourage clinics to use them not only in their treatment programs but also in the prevention of suicidal behavior.

From decision tree no 3, which was chosen to demonstrate this point, it was possible to distinguish factors using a limited but sufficient number of questions to differentiate the group without suicide risk at a specific moment in time. Among the answers that place patients in the “not at risk group,” we found not having thought about taking one’s life, feeling good about oneself (whether one succeeds or fails), not feeling empty inside, frequently feeling like a happy person and being satisfied with life, in addition to being highly concerned with what you receive from others. It has been proven that a feeling of satisfaction regarding one’s own capabilities and caring for others (while maintaining the boundaries of autonomy and social support) are experiences that generate psychological well-being and provide resources for development in life (53). These elements of self-worth and interpersonal relationships can be reinforced through psychotherapeutic intervention to promote and develop the patient’s resources, including boosting them and adopting them for prevention.

Some of the answers that placed patients in a suicide risk configuration included having thought about taking one’s life, having frequently experienced headaches, having felt dissatisfied or not very satisfied with life recently, feeling empty inside at times, and not feeling like a happy person. Factors relating to a depressive lifestyle with their dependent and self-critical style also stand out about being frightened when one feels alone, not worrying a great deal about what relationships with others can offer (54), and not feeling good about oneself whether one succeeds or fails, in addition to not thinking that feeling stable or fearing the act of suicide itself and an absence of reasons to die are significant reasons not to commit suicide (55).

This risk configuration is consistent with the hypothesis that having thoughts about taking one’s life are potentially threatening if they are present in an intense and generalized manner (53). If this state is accompanied by the unbearableness of emotional discomfort alongside difficulties with finding adaptive solutions to adverse situations (56), it becomes a risk factor in which a suicide attempt appears as an escape mechanism from a state that is experienced as intolerable (57). For their part, fears of being alone or of being rejected are often associated with a struggle between attempting to be autonomous and being psychologically dependent on others, creating conflicting relationships that create discomfort and dysfunctionality (58).

There are also reasons to carry on living even when a person is going through a painful, demanding, or overwhelming situation. These findings are consistent with those published in the literature with regard to reasons that might be powerful protectors against suicide (1, 59) and might principally be associated with concern for family and a confidence in one’s ability to face problems (24, 30, 60, 61).

This limited group of variables will enable the development of an assessment tool to track and detect suicide risk. The assessment of a small number of variables in time, which can be quickly and frequently applied and then evaluated by DM techniques, will enable us to recognize suicide risk behavior over time. Being able to detect the moment of psychological vulnerability, which differs for each patient, and its subsequent psychotherapeutic intervention may not only assist in alleviating the condition but also in preventing the risk of suicide. These interventions may be directed to reinforcing psychological well-being, feelings of self-worth and RFL.

It is important to mention that one of the limitations is that this study was based, for the most part, on patients suffering from mood disorders, which enabled us to control for the psychiatric diagnosis variable. However, these results cannot be generalized to other disorders associated with suicidal behavior, such as psychotic disorders or eating disorders, substance dependency disorders, or cognitive disorders (1, 62). The fact that a small number of the patients who were invited to participate in this research declined to do so, even though they met the inclusion criteria, is also considered a limitation. These individuals were not taken into account and are not represented in the results.

It is possible that our results could be applied to a subgroup of subjects. We know that a significant proportion of suicides occur without the subject ever having seen a doctor (63). Our aim is to intervene for the group that could benefit from intervention. Like other conditions, such as obesity and metabolic diseases, therapeutic options often do not enable the treatment of all the subjects, rather they focus on groups of subjects who are open to receiving them. Developing a strategy for those that might benefit them is always a good option in an area with so many different needs. As we have mentioned, the usefulness of these results is limited to a population, and it would doubtlessly be extremely interesting to see how this model behaved for a different population.

We feel it is important to mention that the measurements taken were cross-sectional and show the state of the patient at a precise moment. The expression of these characteristics identifies a particular state surrounding suicidal behavior and has allowed us to determine what factors might be relieved and which could be reinforced in that specific moment of time. It will be necessary for future studies to evaluate the trajectory of these configurations of factors with regard the evolution of suicide risk.

This could be carried out through longitudinal studies that would allow us to shed greater light on the evolution of suicide risk (64). This model could subsequently be developed using an assessment tool that would make use of an artificial intelligence methodology to allow for automated learning, generating a model for detecting suicide risk that would update per the trajectory of patient assessments. It would be possible to do this using a smaller quantity of questions than that used in the original study, allowing for quicker application.

New studies that distinguish between protective factors and suicide risk factors, as well as their configurations, will be necessary. It would be of great interest to reach a better understanding of the state of psychological discomfort that is experienced in an acute fashion, the loss of a sense of self, and reasons for staying alive despite adversity.

## Ethics Statement

This study was carried out in accordance with the recommendations of Comité Ético Científico from Medicin Faculty of Pontificia Universidad Católica de Chile with written informed consent from all subjects. All subjects gave written informed consent in accordance with the Declaration of Helsinki. The protocol was approved by the Comité Ético Científico de la Facultad de Medicina de la Pontificia Universidad Católica de Chile.

## Author Contributions

The authors listed bellow have contributed as follows: SM, JB, and OE made substantial contributions to the conception of the work and interpretation of data for the work; drafting the work and revising it critically for important intellectual content; and final approval of the version to be published and agreed to be accountable for all aspects of the work in ensuring that questions related to the accuracy or integrity of any part of the work are appropriately investigated and resolved. FG and AO made substantial contribution to the analysis of data for the work; revising it critically for important intellectual content; and final approval of the version to be published and agreed to be accountable for all aspects of the work in ensuring that questions related to the accuracy or integrity of any part of the work are appropriately investigated and resolved. CM, MM, RF. CN, TS, and AT made substantial contribution to the acquisition and interpretation of data for the work; revising it critically for important intellectual content; and final approval of the version to be published and agreed to be accountable for all aspects of the work.

## Conflict of Interest Statement

The authors declare that the research was conducted in the absence of any commercial or financial relationships that could be construed as a potential conflict of interest.
